# A Complicated Case of Parturition

**Published:** 1893-02

**Authors:** E. Bowman

**Affiliations:** Glasgow


### SELECTIONS.

### A COMPLICATED CASE OF PARTURITION.

By E. Bowman, M.R.C.V.S., Glasgow.

Some time ago I was asked by a gentleman to look at a handsome boarhound bitch he had recently imported from Germany, which was pregnant, and according to his calculations should have whelped about a week before I saw her.

Although she appeared somewhat restless, I saw nothing to indicate that there would be any difficulty in parturition. On examination, the vulva was found to be considerably swollen and lax, and I could easily insert my hand so as to feel the os uteri, which was freely dilated, but so far could not detect any pup in the opening. I gave it as my opinion that there was no cause for alarm and that probably she would go on alright, and directed that a small dose of castor-oil be given, that she be fed on warm sloppy food, and that no strangers be allowed to visit her; and asked to be informed if anything went wrong. I heard no more for two days, when, meeting the owner, he informed me that the bitch had given birth to eleven puppies, six alive and five dead or dying shortly after birth, and that so far she seemed all right. Consequently I was somewhat surprised on coming home late next night to hear that the owner had called twice, requesting my presence, as the animal seemed to have become suddenly very ill, and was making violent efforts to get rid of something from the bowels. She had refused all food and drink since the evening before, and two of the puppies had died that day.

On reaching the place, I found the animal (by this time 11:30 P.M.) quite paralyzed and somewhat comatose, while the suppression of milk secretion was almost complete; on attempting to turn her about for examination, she gave evidence of much pain. From the history of her behavior I was at first inclined to the belief that there was still a dead and decaying fœtus in the womb which had given rise to septic conditions, but examination rather tended to disprove this, as the os had nearly closed, and the vulva almost attained its normal condition, while with the exception of a very little yellowish discharge there was no indication of any abnormal conditions thereat.

On further examination externally, a hard mass could be felt in the abdominal region; this, taken with the fact that the patient had passed nothing from the bowels for some time with the exception of a foul-smelling watery discharge, led me to conclude there was some obstruction in the bowels. As the animal seemed so weak I had administered to her a dose of brandy, eggs and ammonia; this, although given in small quantity and carefully, was always rejected soon after being given, as was also a dose of castor-oil which was given her. In the intervals of giving the medicine I also twice injected enemata of soap and water with which was mixed a proportion of castor-oil, and while doing so was somewhat astonished at the abnormal quantity which was so easily injected and retained for some time in the bowel. On its return, I observed that on both occasions there was mixed with the injection a quantity of hairs of a greenish color, exactly the same as her own coat, and I was forced to the conclusion that the bitch had either devoured one of her puppies while in the act of parturition, or had subsequently swallowed one of the dead ones which were thrown on to an ashpit in the garden where she was. As the patient was considerably exhausted by this time, I deemed it wise to give her some time to recover, so having her carefully covered up (the puppies had previously been removed from her), she was left for some time. On returning about 4 A.M., she was found to be quite unable to move, and while preparing another medicated injection, a small dose of ammonia, etc., as before was given her; this again was soon after rejected, and before any further steps could be taken she suddenly expired without a change taking place.

The same forenoon I made a post-mortem examination, with the following result: The uterus was found to be twisted on itself, forming two sacs, each one of which contained the half of a fully developed fœtus connected to its fellow through the twist by the skin only; the anterior sac was adherent to the bowel, while at the point of adherence there was a complete perforation through both bowel and uterus, thus accounting for the hairs which were expelled with the injections. With the exception of this lesion, there was nothing either in the stomach or bowels, which were otherwise healthy, and behind the posterior sac the uterus was quite contracted in its entirety. All the other organs appeared to the naked eye quite healthy.

The case was interesting and instructive to me, and I give it in the hope that it may be useful to others. I may mention that a foster-mother was got for the puppies still alive, but that they all died, one after the other, within a week of the mother's death, with symptoms of persistent diarrhœa, this being due I presumed to the conditions of the mother's milk during the time they were allowed to suckle her, as every care was taken of them otherwise. —*The Veterinary Record.*

